# Two Separate Small and Large Ischemic Bowel Events Secondary to Sigmoid Adenocarcinoma: A Case Report

**DOI:** 10.1055/s-0044-1800978

**Published:** 2024-12-20

**Authors:** Alamir-Noureddine AlAyoubi, Souad Ghattas, Hani Maalouf, Georges Chahine, Kiril Kiriakos, Mirna Fares

**Affiliations:** 1 Department of Internal Medicine, Pulmonary Medicine and Critical Care Medicine, Mount Lebanon Hospital University Medical Center, University of Balamand, Beirut, Lebanon; 2 Department of General Surgery, Mount Lebanon Hospital University Medical Center, University of Balamand, Beirut, Lebanon; 3 Division of Critical Care, Department of Pulmonary Medicine and Critical Care Medicine, Mount Lebanon Hospital, University of Balamand, Beirut, Lebanon

**Keywords:** sigmoid adenocarcinoma, acute necrotizing colitis, obstructive colitis, ischemic colitis, small bowels

## Abstract

Colonic obstruction is reported in 85% of emergency colorectal surgery for cancer. Colonic ischemia, however, is a rare entity and is found in 5% of these emergency cases. We herein present the case of a 72-year-old man presenting with signs and symptoms of obstruction and was found to have an obstructive sigmoid cancer. A first urgent laparotomy showed small bowel ischemia, for which small bowel resection and large bowel decompression were done without tumor resection. Postoperatively, the patient transiently improved, then deteriorated in few days, and a second urgent laparotomy showed a large bowel ischemia proximal to the mass. The treatment for patients with colon cancer with suspected colonic ischemia should be emergency laparotomy with long abdominal incision, carefully exploring the whole length of the small and large bowel. Despite large bowel decompression, a secondary colonic ischemic event should be suspected in case of deterioration.

## Introduction

Obstructive colonic cancer is a common presentation of colonic cancer, and it is the major presenting symptom in 8 to 29% of patients with colorectal cancer (CRC), and 85% of emergency colorectal surgery for cancer is for colonic obstruction. However, obstructive colitis (OC), or extensive ischemic colitis, proximally to an obstructive colonic cancer is an uncommon condition. 1 2 In addition, obstructive CRC may be complicated by perforation, electrolyte disturbance, bacterial translocation, and sepsis. 1 OC refers to ulcero-inflammatory lesions occurring in the colon proximal to a completely or partially obstructing lesion. This is in contrast to true ulcerative colitis complicated by colon cancer, where there are inflammatory lesions proximal and distal to the neoplasm. 3 Rarely, OC can progress to acute necrotizing colitis. Only few cases of acute necrotizing colitis due to colon cancer are reported in the literature. 4

In this case report, we present a 72-year-old man who manifested two separate ischemic bowel events secondary to an obstructing sigmoid adenocarcinoma. The first event was small bowel necrosis; the second event was large bowel ischemia that occurred few days later despite colonic decompression. To the best of our knowledge, there is no reported similar case in the literature.

## Case Report

**Fig. 1:**
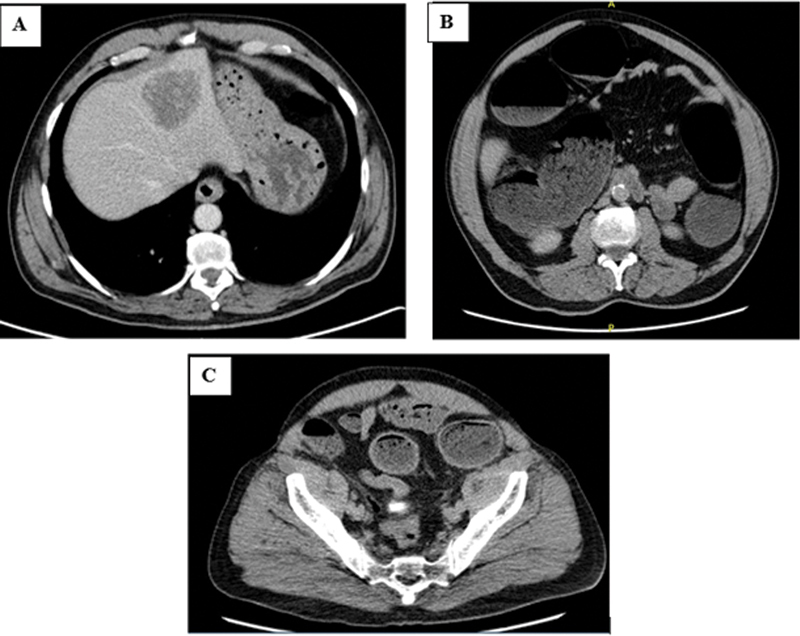
Abdominal enhanced computed tomography scan showing: ( **A** ) highly suspicious intrahepatic mass; ( **B** ) diffuse dilatation of the large bowels; ( **C** ) transition zone in the proximal sigmoid showing mild wall thickening with mild fat stranding raising the suspicion of a malignant lesion.

A 72-year-old male patient with a history of smoking (>100 pack years), coronary artery disease, hypertension, dyslipidemia, diabetes mellitus type 2, and a family history of colon cancer (first-degree relative, age of onset >50 years old) presented for 2 days history of gradually worsening abdominal pain and distension, associated with nausea, vomiting, and constipation of several days duration. He had a 2-months history of gradual weight loss reaching 10 kg, poor appetite, abdominal bloating, and change in bowel habits with decreased stool caliber. His physical examination on admission revealed: blood pressure 123/68 mmHg, pulse 105 bpm, respiratory rate 17/min, temperature 37.3°C, oxygen saturation 97%, positive bowel sounds, distended abdomen with diffuse tenderness, and no guarding or rebound tenderness. Laboratory results showed increased inflammatory markers: leukocyte count 20,500 cells/μL, 85% neutrophils, C-reactive protein 1 mg/dL initially, hemoglobin 17.3 mg/dL, blood urea nitrogen 20 mg/dL, creatinine 0.79 mg/dL, lipase 25 u/L, and normal electrolyte levels. Abdominal pelvis enhanced computed tomography (CT) showed diffuse dilatation of the large bowels proximal to a transition zone in the proximal sigmoid measuring around 3 cm in length, mild wall thickening, and mild fat stranding raising the suspicion of a malignant lesion. A highly suspicious intrahepatic mass was also seen ( Fig. 1 ).

**Fig. 2:**
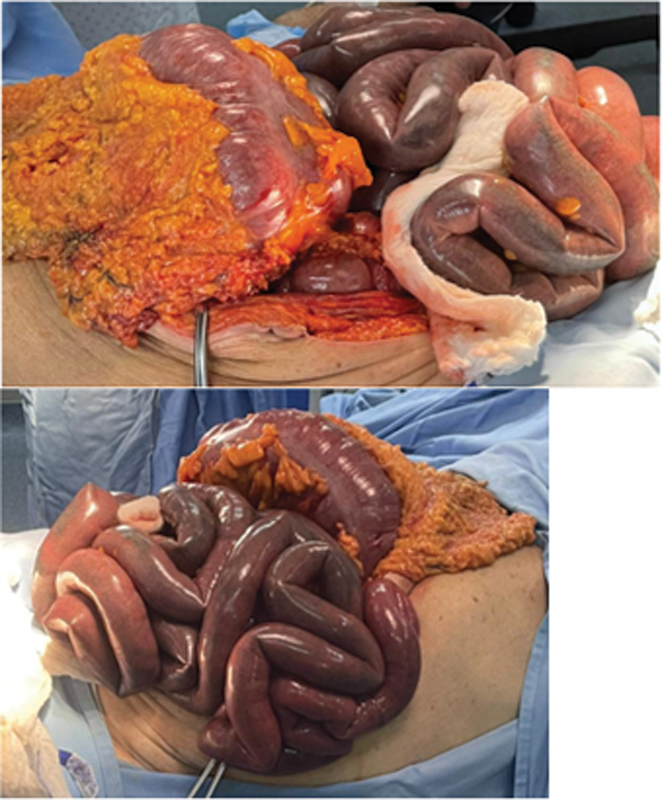
Macroscopic view of the dilated large bowels and necrotic small bowels.

Diagnosis of obstructive sigmoid tumor was done. A nasogastric tube was placed, intravenous (IV) hydration and antibiotics started. Rectal tube was not attempted. The patient deteriorated few hours later, with worsening abdominal pain and increased distension, and emergency laparotomy was decided. After induction, the patient became unstable and was started on vasopressors. Intraoperative findings were sigmoid colon severe stenosis, proximal dilation without perforation, and mild ascites. The small bowels were dilated proximally and black colored from the ileocecal valve up to 2 meters, proximal to the terminal ileum, suggesting acute ischemia without major vessel occlusion. The colon was dilated but viable ( Fig. 2 ).

**Fig. 3:**
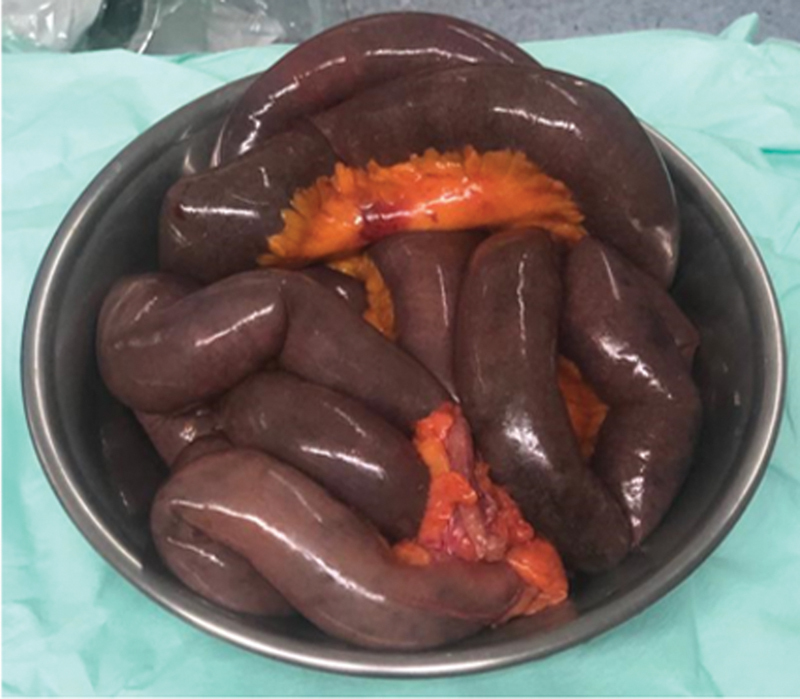
Specimen removed.

A Pezzer tube was inserted in the cecum to drain and decompress the colon. We performed a resection of 2 meters of ischemic small bowel from the ileocecal valve including the appendix and an end ileostomy was created ( Fig. 3 ). Omental and peritoneal lesions were also biopsied and sent to pathology. Final pathology result showed ischemic enteritis, mostly mucosal, with metastatic deposit of adenocarcinoma on proximal appendiceal serosa. Both surgical margins showed ischemic mucosa and transmural inflammation with edematous yet viable submucosa and muscularis propria. Omental and peritoneal lesion returned positive for metastatic adenocarcinoma consistent with the primary tumor of colonic origin.

Postoperatively, the patient was transferred to the incentive care unit, intubated, ventilated, sedated, on vasopressors, and on broad-spectrum IV antibiotics.

**Fig. 4:**
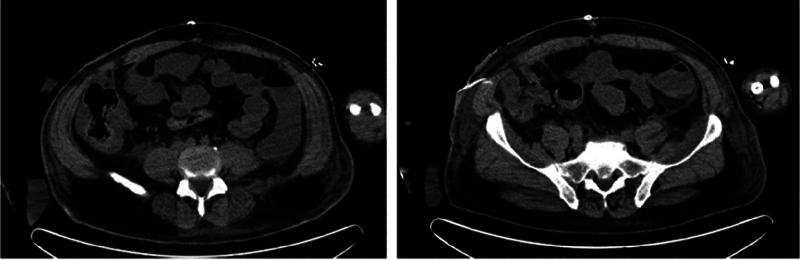
Nonenhanced abdominal computed tomography scan showing right colitis.

The patient's general condition was controlled initially, on minimal vasopressors dose. On postoperative day 5, an abdomen pelvis CT scan without enhancement was done for persistent fever, worsening acute kidney injury (AKI), and increasing Creatine phosphokinase (CPK) level to 7,363 IU/L. CT scan showed findings of right colitis of infectious or ischemic origin that cannot be determined due to lack of IV contrast ( Fig. 4 ). IV antibiotics were empirically escalated.

**Fig. 5:**
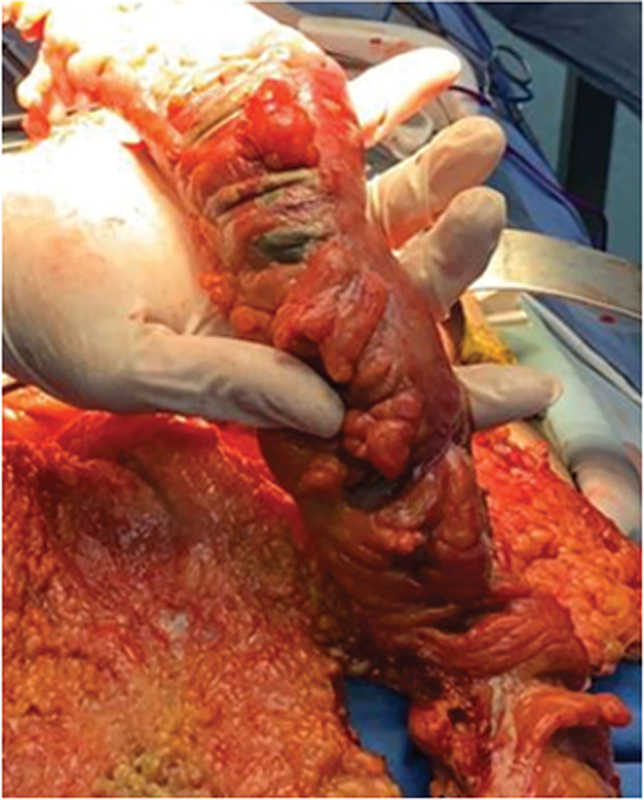
Colonic ischemia proximal to the sigmoid mass.

On postoperative day 6, patient was still febrile with temperature at 39.5°C, now anuric, volume overloaded, creatinine increased to 10.02 mg/dL, and CPK to 117,66 IU/L. Decision was taken to go for urgent dialysis for volume overload due to renal failure secondary to rhabdomyolysis, followed by emergency exploratory laparotomy that showed an ischemic descending colon proximal to the sigmoid mass. Total colectomy (sparing of the rectal vault for possible later anastomosis) was done ( Fig. 5 ). Pathology results reported pT3N0M1c, showing a 2 × 2 cm moderately differentiated adenocarcinoma of the sigmoid colon, completely obstructive with invasion of the serosa, rare vascular emboli, no metastatic lymph nodes (0 out of 16), and ischemia of all the colon with neoplastic thrombosis of the inferior mesenteric vein.

After the second surgery, endotracheal extubation was delayed for 1 week due to recurrent episodes of desaturation secondary to pneumonia-related mucous plugs. Abdominal cultures grew *Enterococcus faecium* , *Escherichia coli* , *Candida albicans* , and *Stenotrophomonas maltophilia* . His infected abdominal fluid was continuously drained in the surgical drain bag, and the infection was treated with wide antibiotics and antifungal coverages. Rectal pouch leakage was diagnosed on imaging fluoroscopy and abdomen-pelvis CT scan. The contrast was identified from the rectal vault surgical clip and up to the right anterolateral draining tube in the abdomen. Management was conservative and the pouch resolved with no surgical intervention. AKI improved after resolution of septic shock and rhabdomyolysis. Gradually, the patient improved, was weaned off pressors and mechanical ventilation, extubated, switched to specialized PO diet for short bowel syndrome, and transferred to a regular floor. On postoperative day 37 from the first operation, he was discharged home on daily Loperamide and Cholestyramine, with close follow-up, vitamins replenishment, good hydration, and low carbohydrate diet for short bowel syndrome.

## Discussion

CRC is the third most common tumor in men and the second in women, accounting for 10% of all tumor types worldwide. CRC is the fourth most common cancer-related cause of death. The mortality rate is 15–20/100,000 in males and 9–14/100,000 in females. 5 6

In total, 20% of patients with CRC are diagnosed with colonic obstruction, mostly on the left side. 5 Colonic obstruction may lead to multiple complications such as perforation, electrolyte disturbances, sepsis from bacterial translocation, OC, and bowel ischemia. 2

In addition, 0.3 to 3.1% of CRCs are associated with OC. 7 OC is defined as a nonspecific inflammatory lesion of the colon, such as erosion and ulceration, proximal to a completely or partially obstructive lesion. It can become fulminant, which is then known as acute necrotizing colitis. 8

In patients with acute colonic obstruction due to malignancy, there are different strategies to decompress colonic obstruction. It can be with nonsurgical approaches such as balloon dilatation, or placement of a rectal tube, or with a surgical approach such as low hole enterostomy and resection of the tumor with or without colostomy. 2 In some cases, resection of the cancer may not be possible because of concomitant medical disease or hemodynamic instability. 9 In our case, due to rapid deterioration of the patient, surgical management was the best option.

For left colon obstruction, two types of surgical approaches can be used. The first is synchronous treatment of carcinoma and obstruction by primary anastomosis. The second is Hartmann procedure, which is the treatment of the obstruction by staged resection. The benefits of staged operation are less surgical trauma, which is significant in patients whose general condition is precarious, and reduction of the risk of contamination due to unprepared bowels. 2 Our patient was hemodynamically unstable post-induction and he was started on vasopressors. We decided to go for a staged operation to reduce operating time and to prevent short bowel syndrome and extensive ileal resection.

Malignant large bowel obstruction can be complicated by an ischemic episode in 5% of the cases. 7 Various etiologies were suggested for this condition: hypovolemic states, systemic diseases like vasculitis and hypercoagulable states, mechanical obstruction, atherosclerosis, bacterial proliferation, therapeutic drug effect including pressors use, and infection. 9 Our patient had multiple factors from these suggested etiologies: hypovolemic states, hypercoagulable states due to malignancies, mechanical obstruction, and bacterial proliferation in the initial ischemic episode. Therapeutic drug effects including pressors use and infection were added on the subsequent ischemic events.

To further explain these possible causes, colon distension from malignant colon obstruction may reduce the blood supply to the bowel wall leading to hypoperfusion and ischemia and colonic gangrene. When endoluminal pressure exceeds 35 cmH _2_ O, intramural perfusion decreases. Once the endoluminal pressure is sustained over 40 cmH _2_ O, there will be irreversible ischemic lesions of the mucosa. The ischemia affects predominantly the hypoxia-sensitive mucosal and submucosal layers. Creation of a decompressed colostomy in malignant colon obstruction can help with the elevated intra-luminal pressure. 2

Earlier surgical intervention may have mitigated the progression to secondary ischemic events in the first presentation considering hypovolemic states, mechanical obstruction, and bacterial proliferation as possible causes of the first ischemic event. But despite decompression and hemodynamic support, our patient has a second episode of distal colon ischemia.

First, histopathologically, OC is characterized by either an erosion or a shallow ulcer confined to the mucosa or submucosa. The most common microscopic findings of OC are necrosis of the mucosa with denudation of epithelial cells, hemorrhage, congestion, and prominent neutrophilic infiltration. The serosa and muscular layer better resist the pressure and preserve their normal aspect. In many cases, the diagnosis is made at exploratory laparotomy. 9 10 Therefore, even severe ischemic lesions may occasionally be difficult to notice in the course of laparotomy. Frequently, the colon has a normal appearance during the surgery. If the surgeon does not have a clinical suspicion of OC, the anastomosis is made through the involved segment of colon with consequent complications, up to 25%. Complications include peritonitis, perforation, bleeding of the remained ulcerative lesion, and breakdown of anastomoses made through involved segments of colon. Awareness of the features and incidence of OC should help physicians avoid these diagnostic and therapeutic problems by applying a thorough approach while inspecting the mucosal and submucosal layers of the bowels. 11

Second, bacterial proliferation in the obstructed bowel may lead to massive bacterial reflux into the ileum from the colon that may cause capillary vasoconstriction of the bowels, causing ischemia. Hypoxia stimulates the germination of spores and bacterial growth, which progresses rapidly to produce exotoxins that destroy the surrounding tissue, leading to rapid spread of the disease. 7

Third, our patient had multiples comorbidities that can result in atherosclerosis and chronic ischemia of the bowels. 7 He was elderly, hypertensive, diabetic, dyslipidemic, and had coronary artery disease.

The use of vasopressors can be an additional factor, decreasing blood supply to the already compromised circulation.

Moreover, colonic ischemia is difficult to diagnose preoperatively due to its occasionally mild and transient clinical presentation, and the symptoms are not specific. The most common presentation is the obstructive syndrome associated with peritonitis. Common clinical symptoms are abdominal pain, tenderness, constipation or watery diarrhea, hematochezia, nausea, vomiting, and fever. 3

It was difficult to diagnose colonic ischemia in this patient suspected to have colonic obstruction due to a distal colon cancer. In the initial presentation of the patient, we opted for emergency surgery because our patient was deteriorating rapidly. Enhanced CT scan did not change the final management and might simply have delayed the operation. Relying on radiologic findings to diagnose ischemia can be misleading in most cases. In a series of seven patients reported by Chang et al 1 , the radiologic findings of the abdominal CT of the six cases were compatible with colon or rectal cancer and distention of the proximal bowel without ischemic changes. There was only one case where proximal colonic ischemia was diagnosed with abdominal CT prior to surgery. 3

Of note, the surgeon should pay attention to whether the dilated colon is viable or not before creating an anastomosis. 8 During the first operation, we confirmed that the colon proximal to the obstruction was viable. A staged operation was done to reduce surgical trauma. Resection of all ischemic small bowels and cecal decompression were performed. However, he deteriorated again after 5 days, with rhabdomyolysis and septic shock. Lack of IV contrast made our diagnosis of colonic ischemia difficult on repeat CT scan, yet we had a high clinical suspicion for bowel ischemia. Our suspicion was confirmed by the findings of the second operation.

Equally important, the gross finding of OC is large, irregular ulceration with mucosa covered by a hemorrhagic and purulent exudate. The margins of the ulcers are frequently sharply defined with intact intervening mucosa. The wall of the diseased colon is usually thickened, stiff, and friable. The mucosa of the colon distal to the malignant lesion is normal grossly and microscopically. Frequently, 2 to 6 cm proximally to the carcinoma is usually free of ulceration and inflammation. The rest of the proximal colon will have varying degrees of inflammation and ulceration. 3 Most necrotizing enterocolitis involves a single bowel segment (50%), but some cases may involve multiple segments. The terminal ileum is the most common site of necrotizing enterocolitis, followed by the large intestine. About 44% of necrotizing enterocolitis cases occur in both the large and small intestines. Yet, this case is the only one reported to have a stepwise presentation of small and then large bowel ischemia, to the best of our knowledge. Pan-necrotizing enterocolitis or necrotizing enterocolitis totalis is fulminant necrotizing enterocolitis characterized by necrosis of more than 75% of the intestine. As many as 19% of all cases of necrotizing enterocolitis treated with surgery and necrotizing enterocolitis causing death were pan-necrotizing enterocolitis. 9

## Conclusion

OC is an uncommon yet lethal condition that is confined to the mucosa or submucosa making it difficult to be identified during the laparotomy as the serosal surface of the colon may be normal. The surgeon must examine the removed colonic segment to exclude an ischemic process in the area of the anastomosis. Hence, the treatment for patients with suspected colonic obstruction should be an emergency operation with long abdominal incision to allow careful and detailed inspection of the whole small and large bowels.

Because the involved segments of the colon appear externally normal during laparotomy, it is important to open the resected bowel in the operative field and inspect the mucosal surface of the dilated and thickened bowel to exclude any inflammatory or ischemic process.

## References

[1] Chang H K Min B S Ko Y T Obstructive colitis proximal to obstructive colorectal carcinoma Asian J Surg 2009 32 01 26 32 19321399 10.1016/S1015-9584(09)60005-1

[2] Huang W S Liu K W Lin P Y Hsieh C C Wang J Y Delayed ischemic gangrene change of distal limb despite optimal decompressed colostomy constructed in obstructed sigmoid colon cancer: a case report World J Gastroenterol 2006 12 06 993 995 16521237 10.3748/wjg.v12.i6.993 PMC4066174

[3] Moldovan R Vlad N Curca G Total necrotizing colitis proximal to obstructive left colon cancer: case report and literature review Chirurgia (Bucur) 2013 108 03 396 399 23790791

[4] Moriwaki Y Sugiyama M Toyoda H Lethal obstructive colitis: how and when patients with colonic obstruction should be prevented from falling into a lethal condition Hepatogastroenterology 2009 56(91–92):659 662 19621675

[5] ESMO Guidelines Committee. Electronic address: clinicalguidelines@esmo.org Argilés G Tabernero J Labianca R Localised colon cancer: ESMO Clinical Practice Guidelines for diagnosis, treatment and follow-up Ann Oncol 2020 31 10 1291 1305 32702383 10.1016/j.annonc.2020.06.022

[6] AFC (French Surgical Association) Working Group Manceau G Sabbagh C Mege D Colon sparing resection versus extended colectomy for left-sided obstructing colon cancer with caecal ischaemia or perforation: a nationwide study from the French Surgical Association Colorectal Dis 2020 22 10 1304 1313 32368856 10.1111/codi.15111

[7] Matsunaga H Shida D Kamesaki M Hamabe Y Acute necrotizing colitis due to sigmoid colon cancer World J Surg Oncol 2014 12 19 24460766 10.1186/1477-7819-12-19 PMC3906958

[8] Tsai M H Yang Y C Leu F J Obstructive colitis proximal to partially obstructive colonic carcinoma: a case report and review of the literature Int J Colorectal Dis 2004 19 03 268 272 14704804 10.1007/s00384-003-0558-0

[9] Kimura J Lefor A K Kubota T Colonic ischemia mimicking obstruction due to sigmoid colon cancer: a case report Int J Surg Case Rep 2018 46 38 40 29679809 10.1016/j.ijscr.2018.04.008 PMC6000759

[10] Ohta K Kijima T Tokuhisa Y Morita K A case of ileus due to colon cancer with non-occlusive mesenteric ischemia [in Japanese] Gan To Kagaku Ryoho 2020 47 04 643 645 32389970

[11] Kimura J Lefor A K Kubota T Colonic ischemia mimicking obstruction due to sigmoid colon cancer: A case report Int J Surg Case Rep 2018 46 38 40 29679809 10.1016/j.ijscr.2018.04.008 PMC6000759

